# Expression of PD1 and PDL1 as immune-checkpoint inhibitors in mantle cell lymphoma

**DOI:** 10.1186/s12885-022-09803-x

**Published:** 2022-08-03

**Authors:** Fereshteh Ameli, Elham Shajareh, Maral Mokhtari, Farid Kosari

**Affiliations:** 1 Department of Pathology, Cancer Institute, Imam Khomeini Hospital Complex, Tehran University of Medical Science, Tehran, Iran; 2 Department of pathology, Shiraz University of Medical Science, Shiraz, Iran; 3 Department of Pathology, Shariati Hospital, Tehran University of Medical Science, Tehran, Iran

**Keywords:** Lymphoma, Mantle-Cell, Programmed Cell Death 1 Receptor, Programmed Cell Death 1 Ligand 2 Protein

## Abstract

**Background:**

Mantle cell lymphoma (MCL) has remained incurable in most patients. The expression of PD-L1 as a prognostic and predictive marker has not been fully evaluated in MCL. The current study aimed to determine PD-1/PD-L1 expression in MCL specimens and its significance as an immune check point inhibitor.

**Methods:**

This retrospective study was conducted on the formalin-fixed paraffin-embedded blocks of 79 confirmed MCL patients based on immunohistochemistry (IHC). The IHC method was used to stain patient samples for PD1 and PDL1. Positive PD-1/PD-L1 expression was defined as moderate to strong or memberanous or memberanous/cytoplasmic staining in at least 5% of tumor and/or 20% of associated immune cells. Tumor aggressiveness was determined based on Ki67 and variant.

**Results:**

The mean age of the patients was 60.08 ± 10.78 years old. Majority of the patients were male. The prevalence of aggressive tumor was 25%. Positive PD1 and PDL1 expression were identified in 12 (15.0%) and 3 (3.8%) of tumor cells, respectively. PD1 and PDL1 were positive in zero (0%) and 7 (8.9%) of background cells, respectively. There was no significant difference in terms of study parameters between positive and negative groups for both PD1 and PDL1 proteins. PD1 tumor cell percentage was negatively correlated with age (*r* = -0.254, *p* = 0.046).

**Conclusion:**

Our results suggest that neither PD-1 nor its ligands represent relevant targets for MCL treatment. Age may impact the efficiency of immune checkpoint inhibitors and could be related to the increased incidence of MCL with age.

## Background

The incidence of lymphomas has increased in recent years and lymphomas now account for 3% of death due to cancer [1]. Mantle cell lymphoma (MCL) is an aggressive subtype of B cell lymphomas that cannot be treated in many cases. It was reported that 6% of non-Hodgkin's lymphomas are MCL.

MCL is usually diagnosed at advanced stages and its prognosis is the worst among B-cell lymphomas [2]. The average survival for MCL is 3 to 5 years [2]. MCL treatment has always been a challenge for physicians. The current treatments for MCL include Rituximab, Bruton’s tyrosine kinase (BTK) inhibitors, BCL2 inhibitors, and stem cell transplantation. However, these treatments were found to be ineffective in the treatment of MCL relapse and refractory MCL [3–5].

Immune-based treatment strategies, including immune checkpoint inhibition, have recently been proposed as treatment options in B-cell-derived lymphomas. Checkpoint inhibition has shown promising results in the treatment of specific lymphoma subtypes like Hodgkin lymphoma. Nevertheless, the effectiveness and benefits of checkpoint inhibition in the treatment of other entities are still being investigated [6].

Many immune checkpoint molecules, including Programmed Death 1 (PD-1) and its ligands PD-L1 and PD-L2; Cytotoxic T Lymphocyte Activator 4 (CTLA-4); Lymphocyte Activation Gene 3 (LAG-3), and CD200, among others, involve in tumor immunology [7]. Unlike T-cells, programmed death 1 (PD-1), programmed death ligand 1 (PD-L1), CTLA-4, and LAG-3 are rarely expressed in other immune effector cells [8–12]. Ligation of these proteins reduces immune cell activation and cytotoxicity, proliferation, and cytokine production [8–12]. PD-L1 expression is a predictive biomarker for the treatment of solid malignancies and has been routinely used for this purpose in clinical practice [13–16].

Interaction between PD-L1 ligands on tumor cells and lymphocyte PD-1 suppresses immune response; therefore, PD-L1 expression in tumor cells indicates that the tumor cells are capable of immune evasion [17]. Therefore, it is important to evaluate PD-1 within tumor-infiltrating lymphocytes in order to understand the tumor-immune interaction [17]. Evaluation of PD-L1 expression through immunohistochemistry (IHC) can predict the response to checkpoint inhibitors and can be used to qualify patients for immune checkpoint inhibitors [18].

Furthermore, the effects of various checkpoint inhibitors have been studied on multiple myeloma, chronic lymphocytic leukemia, acute myeloid leukemia, myelodysplastic syndrome, diffuse large B-cell lymphoma, follicular lymphoma, and cutaneous T-cell lymphoma [19]. For instance, Pembrolizumab is an FDA-approved therapy for classic Hodgkin lymphoma [19]. However, there is scarcity of data regarding the expression or function of these molecules in MCL. The findings of the current studies are conflicting and inconclusive in terms of implementing immune checkpoint inhibitors in the treatment of MCL [2, 20–22]. Herein, this study was conducted to investigate the expression of PD1 and PDL1, as immune checkpoint molecules, in MCL using IHC.

## Methods

### Study population

This retrospective-observational study was performed on 79 confirmed MCL patients based on immunohistochemistry (IHC). Specimens were obtained from the archives of surgical pathology departments of Imam Khomeini Hospital Complex and Shariati hospitals, affiliated to Tehran University of Medical Science and Namazee hospital affiliated to Shiraz University of Medical Science between January 2015 and December 2020. Sample collection was based on universal sampling method. Two expert pathologists reviewed all previously evaluated Hematoxylin and Eosin slides to confirm the diagnosis. The paraffin blocks of documented MCL specimens were obtained for immunohistochemistry study. The clinicopathological variables included age at diagnosis, sex, tumor location, leukemic involvement, and ki67 proliferation index as well as tumor variant that were obtained through Laboratory Information System and/or the surgical department records. This study was approved by the Ethics Committee of the Tehran University of Medical Sciences (IR. TUMS. IKHC.REC.1399.468).

### Immunohistochemistry Staining

Mouse monoclonal anti-human PD1/PDL1 Clone SBC-991 IgG1 Isotype (Cat. No: SB-019261, SINA BIOTECH) at a dilution of 1:200 was used for immunohistochemical (IHC) staining. Tonsil tissue was used as control tissue. Briefly, deparaffinized sections were rehydrated and subjected to heat antigen retrieval technique. The standard protocols provided by the manufacturer was used for immunostaining.

Two pathologists (F.A. and E.Sh.) who were blinded to sample identity independently quantified all stains. Percentage of malignant and non-neoplastic background immune cells with positive staining (0% to 100%), intensity of staining (0 = no staining, 1 +  = weak, 2 +  = moderate and 3 +  = strong staining) and primary subcellular localization of positive staining (nuclear, cytoplasmic or cell membrane) were recorded. Previously published criteria for categorizing cases as positive for PD-L1 or PD1 expression in malignant cells were used [23–26].

Malignant cells needed to exhibit 2 + or 3 + membrane or cytoplasmic/membranous staining in > 5% of malignant cells to consider as positive and for the tumor microenvironment, > 20% of nonmalignant cells needed to exhibit positive staining for PD-L1 or PD1 to be categorized as positive.

In case of disagreement in positivity between pathologists, samples were re-evaluated simultaneously by the two pathologists to reach consensus. Data regarding the demographic characteristics, histopathology, and IHC findings were collected using a researcher made checklist and were transferred to an Excel worksheet.

### Statistical analysis

Data analysis was performed using the statistical package for social sciences (SPSS) software version 16 (IBM Inc, Chcago, Il, USA). The Shapiro–Wilk test was used to evaluate the normality distribution of continuous variables. Continuous variables were described using mean and standard deviation. Comparison of continuous variables between groups was performed using the independent t-test. Categorical variables were described using frequency and percentage and were compared between groups using the Fisher exact or Monte Carlo tests. The Spearman correlation coefficient was used to evaluate the correlation between study parameters and PD1 and PDL1 status and the Spearman correlation coefficient (r) and p value were reported for the analysis. Univariate and multivariate binary logistic regression were performed to evaluate the relationship between study parameters and PDL and PDL1 positivity. Logistic regression results were presented using expected Beta (ExpB), 95% confidence interval (CI) for ExpB and p value. Level of statistical significance was considered as *p* < 0.05.

## Results

A total of 79 samples were evaluated in this study. The mean age of the patients was 60.08 ± 10.78 years old. Majority of the patients were male (male: female ration = 4:1). The mean size of the extracted tumors was 1.24 ± 0.64 cm. The most common biopsy location was extranodal (44, 55.6%) followed by nodal in 31 (27.7%) cases. Extra nodal tumors included head and neck (5, 6.5%), gastrointestinal (8, 10.8%), and bone marrow (31, 38.8%). Based on the Ki67 and variant, aggressive tumors were detected in 20 (25%) of the tumors in our study.

**Fig. 1:**
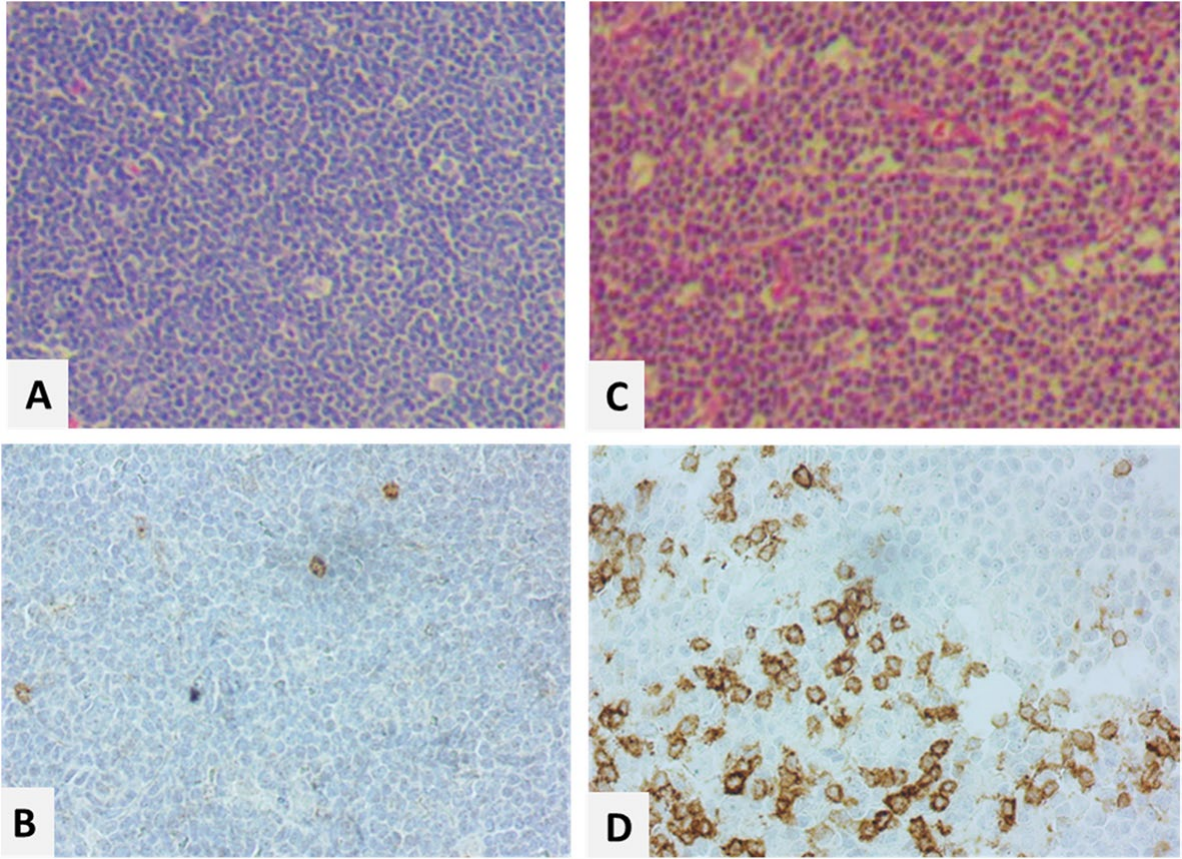
Hematoxylin & Eosin with PD1 immunohistochemical staining images of Mantle cell lymphoma (× 400): A& B: expression of PD1 in a PD1 negative case, C&D: a PD1 positive case with 40% imunoreactivity in tumor cells

**Fig. 2:**
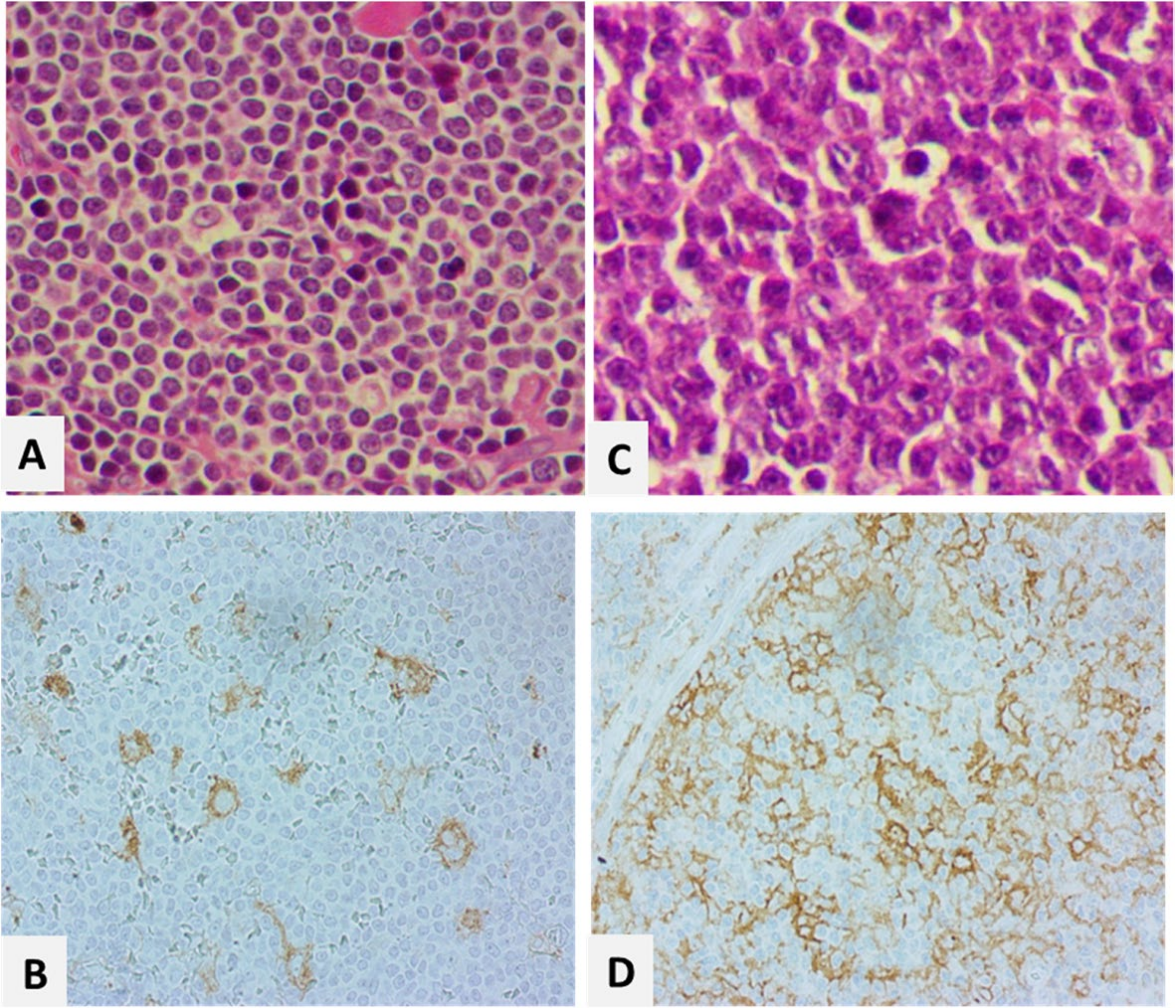
Hematoxylin & Eosin with PDL1 immunohistochemical staining images of Mantle cell lymphoma (× 400): A& B: a PDL1 negative case (< 5%), C&D: a PDL1 positive case with 60% imunoreactivity in tumor cells

The PD1 and PDL1 were positive in 12 (15.0%) and 3 (3.8%) of tumoral cells, respectively. PD1 and PDL1 were positive in zero (0%) and 7 (8.9%) of background cells, respectively (Figs. 1 and 2). Most of the positive cases were from cervical lymph nodes and background immune cells mainly were histiocytes. There was no significant age difference between groups (*p* > 0.05). Comparison of study parameters between PD1 and PDL1 categories among tumoral and background cells are presented in Table 1. There was no significant difference in gender, variant, and Ki67 categories between groups (*p* > 0.05).

**Table 1:** Comparison of study variables between PD1 and PDL1 groups

		Tumoral						Background					
		PD1		p	PDL1		p	PD1		P	PDL1		P
		Negative Frequency (%)	Positive Frequency (%)		Negative Frequency (%)	Positive Frequency (%)		Negative Frequency (%)	Positive Frequency (%)		Negative Frequency (%)	Positive Frequency (%)	
Gender	Male	11 (50.0%)	11 (50.0%)	0.588^a^	37 (92.5%)	3 (7.5%)	0.396^a^	5 (22.7%)	17 (77.3%)	0.180^a^	39 (97.5%)	1 (2.5%)	0.363^a^
	Female	2 (66.7%)	1 (33.3%)		9 (100.0%)	0 (0.0%)		2 (66.7%)	1 (33.3%)		9 (90.0%)	1 (10.0%)	
Variant	Pleomorphic	0 (0.0%)	1 (100.0%)	0.240^a^	3 (100.0%)	0 (0.0%)	0.999^b^	0 (0.0%)	1 (100.0%)	0.999^a^	(100.0%)	0 (0.0%)	0.999^b^
	Classic	16 (59.3%)	11 (40.7%)		57 (95.0%)	3 (5.0%)		9 (33.3%)	18 (66.7%)		60 (96.8%)	2 (3.2%)	
	Blastoid	-	-		3 (100.0%)	0 (0.0%)		-	-		3 (100.0%)	0 (0.0%)	
Ki67	< 30%	1 (25.0%)	3 (75.0%)	0.999^a^	7 (100.0%)	0 (0.0%)	0.523^a^	1 (25.0%)	3 (75.0%)	0.999^a^	7 (87.5%)	1 (12.5%)	0.999^a^
	≥ 30%	4 (36.4%)	7 (63.6%)		12 (80.0%)	3 (20.0%)		2 (18.2%)	9 (81.8%)		14 (93.3%)	1 (6.7%)	

^a^ The Fisher exact test was used for the comparison

^b^ The Monte Carlo test was used for the comparison

There was no significant difference in terms of study parameters between PD1 and PDL1 positive and negative cases. Multivariate relationship between study parameters and PD1 and PDL1 positivity in tumoral and background cells are shown in Table 2. There was no significant relationship between study parameters and positivity of neither the PD1 nor PDL1 markers.

**Table 2:** Multivariate relationship between study parameters and PD1 and PDL1 positivity in tumoral and background cells

Variable		p	Exp(B)	95% C.I.for EXP(B)	
				Lower	Upper
Gender	PD1 positivity in tumor cell	0.999	< 0.001	< 0.001	-
	PDL1 positivity in tumor cells	0.999	< 0.001	< 0.001	-
	PD1 positivity in background cells	0.999	< 0.001	< 0.001	-
	PDL1 positivity in background cells	0.505	0.028	< 0.001	1045.587
Age	PD1 positivity in tumor cell	0.503	0.953	0.829	1.096
	PDL1 positivity in tumor cells	0.389	1.050	0.939	1.174
	PD1 positivity in background cells	0.996	0.011	< 0.001	-
	PDL1 positivity in background cells	0.605	0.885	0.556	1.408
Size	PD1 positivity in tumor cell	0.389	0.421	0.059	3.017
	PDL1 positivity in tumor cells	0.658	0.514	0.027	9.830
	PD1 positivity in background cells	0.966	1.873E + 41	< 0.001	-
	PDL1 positivity in background cells	0.467	< 0.001	< 0.001	3.481E + 15
Aggressive tumor	PD1 positivity in tumor cell	0.999	< 0.001	< 0.001	-
	PDL1 positivity in tumor cells	0.999	289,378,866.6	< 0.001	-
	PD1 positivity in background cells	0.999	633,488,326.857	< 0.001	-
	PDL1 positivity in background cells	0.488	379.467	< 0.001	7,470,964,009

There was a significant negative correlation between PD1 tumor cell percentage and age (*r* = -0.254, *p* = 0.046). This finding indicates that by increase in age, the PD1 tumoral cell percentage decreases.

## Discussion

In tumor microenvironment, the PD-1 immune checkpoint has a crucial role in T cell exhaustion that leads to tumor evasion. Ligands of PD-1, namely programmed death ligand 1/2 (PD-L1/L2), are over-expressed in tumor cells [27]. These ligands affect tumor progression time and survival [27]. Immune checkpoint blockade therapies (ICBTs) that target PD-1 and its ligands (PD-L1/B7-H1/CD274) were reported to have significant clinical benefits and result in durable treatment response in many tumor types [28].

Regardless of its significant effects on cancer treatment, not all patients can benefit from immunotherapy [29, 30]. Therefore, the current concern is to identify reliable biomarkers to be used in the selection of susceptible patients to immunotherapy and at the same time prevent serious toxicities and treatment costs in non-responding patients. Based on the current literature, clinical response to immunotherapy could be identified through IHC based on PDL-1, microsatellite instability (MSI), tumor mutational burden (TMB), T-cell receptor clonality, and the level of T-cell infiltration, as well as the expression of signature genes and peripheral blood biomarkers [31]. However, the currently available single biomarkers have limitations when applied to real-world clinical settings[32].

The presence of these biomarkers, including PD1 and PDL-1, on the surface of tumor cells in MCL was previously evaluated, but the findings of the studies were inconclusive and the sample size of former studies was too small.

The results of the present study revealed that, PD1 immunoreactivity was observed in 15% and 0% of tumoral and background cells, respectively. Positive PDL-1 expression was noted in 3.8% and 8.9% of tumor and background cells, respectively. Most of immune background cells in our study were macrophages. This could be further explained with presence of minimal tumor infiltrating T lymphocytes in the background of mantle cell lymphoma cases. Some studies discouraged the use of immune background cells, including macrophages, as positive cells [33, 34].

Our findings were compatible with the findings of the study by Karalova et al., which showed weak expression of PD1 and PDL1 on B and T cells of MCL cases compared to healthy individuals based on flow cytometry [35]. Menter et al. also reported low or no PDL1 expression in MCL patients based on IHC evaluation [22]. These findings also supported the findings of the current study.

Yang et al. showed that the highest level of PD-L1 expression was observed in diffuse large B-cell lymphoma, followed by small lymphocyte lymphoma, mucosa-associated lymphoid tissue lymphoma, mantle cell lymphoma, while follicular lymphoma had the lowest PD-L1 expression level [36]. These findings suggest that PD-L1 may be associated with lymphoma invasiveness [36].

Few studies have evaluated the immune environment of MCL. Some studies reported that PDL1 expression was low in MCL [22, 35, 37, 38] and that PD1 and PDL1 were not relevant targets for MCL therapy.

In contrast, Harrington et al. in 2019, reported PD-L1 expression in blood samples of six leukemic MCL patients using PCR [39]. This controversy could be related to the difference in the method used for evaluating the expression of these markers. While, some studies examined PD1 and PDL1 expression at the mRNA level, others, including the current study, evaluated gene expression at the membrane protein level. However, Harrington examined only 6 cases at molecular level without specifying that these MCL cases were indolent leukemic variant or aggressive cases with advanced leukemic presentation. Considering the significant findings of the current study, which had a larger sample size, further molecular studies on larger samples are required to draw a conclusion.

Lesokhin et al. evaluated the efficacy and safety of Nivolumab in patients with lymphoma. They found that follicular lymphoma (FL) and diffuse large B cell lymphoma (DLBCL) presented the highest objective response, while other B-cell lymphomas including MCL lacked objective response to treatment. However, in their study, only four patients with MCL were evaluated [26].

Durvalumab is a humanised IgG1-kappa monoclonal antibody that is selective and has high-affinity against PD-L1. Durvalumab was found to be able of reducing tumor growth by 75% in both in vitro and in vivo xenograft studies if accompanied with tumour-reactive human T-cells. This finding indicates the immunological mechanism durvalumab against tumor cells. The findings of animal studies indicate that anti–PD-L1 therapy might have synergistic effects on the antitumor activity of ibrutinib (BTK inhibitor). Combination therapy with durvalumab and ibrutinib was found to be associated with objective response rate( ORR) in a sample of 10 MCL patients. Durvalumab administration combined with rituximab and bendamustine was found to be associated with 88.9% ORR in FL and 30% ORR in DLBCL patients. Furtnermore, monotherapy with durvalumab showed no response in neither of the of FL, MCL or DLBCL patients [6].

Considering the controversial findings of previous studies, combination of biomarkers and using multiplex way algorithms based on artificial intelligence might increase the success rate in selecting immunotherapy susceptible patients[40].

For example, a meta-analysis indicated PD-L1 as a valuable predictive biomarker in immunotherapy in selected tumors. The meta-analysis indicated that monotherapy with PD-1/PD-L1 reduced mortality by 14% in patients with negative PDL-1 findings, which comprise of nearly 10% of the PD-L1 negative cancer patients. Therefore, PD-L1 expression cannot be used as a definit predictor of response to monotherapy with PD-1/PD-L1 in all patients.

Thereby, it is a clinical challenge to predict response to PD-1/PD-L1 blockade immunotherapy. There is a need for studies to assist clinical decision making by identifying factors that affect the strength and duration of response to immunotherapy.

Furthermore, previous studies have reported that different cancers with different PD-L1 expression present different PD-1/PD-L1 monotherapy response. These factors should be used in decision making in patient discussions before they initiating PD-1/PD-L1 blockade immunotherapy[32].

High correlation has been reported between clinical outcomes and both the tumoral and tumor-associated immune cell PD-L1 staining[34].

PD-L1 expression scoring was found to have good-to-excellent reliability of scoring. However, the reliability of immune cell scoring was found to be lower compared to tumor cells. FDA has developed guidelines to evaluate immune cells to determine viable tumor cells in cancer and immune cells, including tumor cells, lymphocytes, and macrophages [33].

Our study used previously described criteria to determine positive lymphoma cases for PD-L1. Due to the better results of PDL1 expression assessment, PD1 immunohistochemistry study is not recommended in selecting patients for checkpoint inhibitors therapy. However, the findings of a previous study indicated that immunotherapy results might be affected by PD1 expression on tumor cells. The study by Xiaodong Wang demonstrated that intrinsic PD-1 receptor was a tumor suppressor that could mediate resistance to PD-1 blockade therapy. This finding requires further affirmative results in other studies [41].

The results of our study did not show any difference in in sex, age and tumor size between positive and negative PD1 and PDL1 groups. No significant correlation was found between prognostic pathological factors such as subgroup and Ki-67 index and PD1 or PDL1 expression.

Another important finding of the present study was the significant negative correlation between the age of patients and the percentage of PD1 tumor cells. In other words, the percentage of PD1-positive tumor cells in MCL patients decreased with age. Karolova et al. reported age-related changes in the expression of PD1 and its ligand (PDL2) in healthy volunteers [35]. These findings are important in identifying patients who may benefit from treatment with PD1 inhibitors. It seems that patient’s age may have a negative effect on the effectiveness of this treatment.

In the current study, PD1 positivity in tumor cells was inversely related to PDL1 positivity in background cells and by increasing PD1 positivity in tumor cells, PDL1 positivity decreased in background cells. Further studies are required to better explain this inverse relationship.

Physiologically, activated B and T cells; macrophages and histiocytes; and dendritic cells express PD1 on their surface [42]. In the current study, PD1 and PDL1 expression was observed mainly on macrophage and histiocytes in the background of tumor.

## Conclusion

The findings of the present study indicated a relatively low immunohistochemical expression for PD1 and PDL1 markers in tumoral and background cells in patients with Mantle cell lymphoma. Therefore, PD1 or PDL1 inhibitors do not seem to be suitable treatment options for immunotherapy in most patients with Mantle cell lymphoma.

It seems that age may have a negative effect on the effectiveness of checkpoint inhibitor therapy. This finding may also explain the increase in the risk of cancer with age due to immune senescence which should be further investigated.

Finally, understanding the interaction between malignant cells, and immune-accompanying cells in tumor microenviroment is mandatory for the purpose of choosing the best treatment option.

## Data Availability

The datasets generated and/or analysed during the current study are not publicly available due to university rules and regulation of data ownership but are available from the corresponding author on reasonable request.

## Abbreviations

MCL: Mantle cell lymphoma
BTK: Bruton’s tyrosine kinase
PD-1: Programmed Death 1
CTLA-4: Cytotoxic T Lymphocyte Activator 4
LAG-3: Lymphocyte Activation Gene 3
PD-L1: Programmed death ligand 1
IHC: Immunohistochemistry
SPSS: Statistical package for social sciences
ExpB: Expected Beta
CI: Confidence interval
